# Thoracic Renal Artery

**DOI:** 10.1055/s-0039-1693987

**Published:** 2019-10-15

**Authors:** Umberto G. Rossi, Gian Andrea Rollandi

**Affiliations:** 1 Department of Diagnostic Imaging, Interventional Radiology Unit, Ente Ospedali Galliera Hospital, Genova, Italy; 2 Department of Diagnostic Imaging, Radiology Unit, Ente Ospedali Galliera Hospital, Genova, Italy

**Keywords:** renal artery, ectopic renal artery, computed tomography, vascular variant, aorta

## Abstract

We report a case of right renal artery originating from the thoracic aorta with normal position of the right kidney. Although this anomaly is rare, vascular, thoracic, and urological surgeons should be aware about this vascular variant before planning their surgical intervention.

A 69-year-old man, with abdominal aortic aneurysm, underwent multidetector computed tomographic (MD-CT) angiography as a diagnostic investigation for possible endovascular aneurysm repair (EVAR). The examination confirmed abdominal aortic aneurysm with a diameter of 5.1 cm. In the scans passing through the lower thorax, a right single ectopic renal artery originating at the level of T12 vertebra was found ( Fig. 1A, B ). In addition, the right single ectopic renal artery was associated with a normal position of the ipsilateral kidney. This anatomical variant did not affect or preclude the subsequent EVAR.

**Fig. 1:**
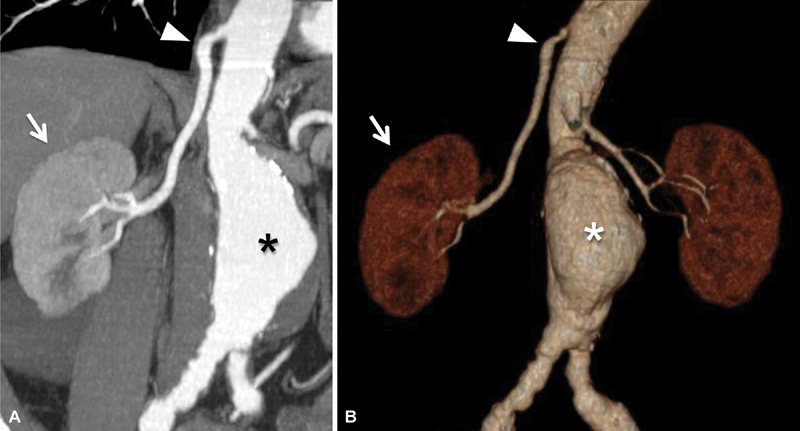
( **A, B** ) Multidetector computed tomographic angiography with ( **A** ) coronal maximum intensity projection and ( **B** ) volume rendering reconstruction technique that demonstrates the presence of the abdominal aortic aneurysm (*) and the normal position of the right kidney (arrow) with a single ectopic renal artery originating from the thoracic aorta (arrowhead).

Knowledge of variations in kidney vascular anatomy, in terms of number and origin, is important for patients who are candidates for EVAR, renal transplantation, renal surgery, and/or endovascular treatment. 1 2 3 4 This is crucial to prevent possible complications or errors.

MD-CT angiography is now the first-line diagnostic method to detect vascular anatomy, including renal anatomy. 1 There are several reports of ectopic renal arteries in the literature. But, to our knowledge, this is only the seventh report of single ectopic renal artery originating in the thorax with normal kidney position. 2 5
